# PRIMA: randomized prospective multicenter non-inferiority study for primary diagnosis of clinically significant *PR*ostate cancer by PSA and MR *IMA*ging—study protocol for a randomized diagnostic accuracy trial

**DOI:** 10.1186/s13063-026-09750-z

**Published:** 2026-04-25

**Authors:** Rouvier Al-Monajjed, Peter Albers, Matthias Boschheidgen, Jan Philipp Radtke, Johanna Droop, Axel Benner, Boris Hadaschik, Gerald Antoch, Lars Schimmöller, the PRIMA Study Group

**Affiliations:** 1 Department of Urology, University Hospital Düsseldorf, Heinrich-Heine-University, Düsseldorf, Germany; 2 Division of Personalized Early Detection of Prostate Cancer (C130), German Cancer Research Center, Heidelberg, Germany; 3 Department of Diagnostic and Interventional Radiology, University Hospital Düsseldorf, Düsseldorf, Germany; 4 Institute for Diagnostic, Interventional Radiology and Nuclear Medicine, Marien Hospital Herne, University Hospital of the Ruhr University, Bochum, Germany; 5 Division of Biostatistics (C060), German Cancer Research Center, Heidelberg, Germany; 6 Department of Urology, University Hospital Essen, University of Duisburg-Essen, Essen, Germany; 7 Department of Radiology, German Cancer Research Center (DKFZ), Heidelberg, Germany; 8 Center for Integrated Oncology Aachen Bonn Cologne Dusseldorf (CIO ABCD), Düsseldorf, Germany

**Keywords:** Prostate cancer, MpMRI, Targeted biopsy, PSA, Diagnostic accuracy, Randomized trial

## Abstract

**Background:**

Diagnostic pathways based on PSA, digital rectal examination (DRE), and systematic biopsy (SB) may miss clinically significant prostate cancer (csPCa) and lead to overdiagnosis of indolent disease. Multiparametric MRI (mpMRI) and MRI-targeted biopsy (TB) improve detection of csPCa; however, the additional diagnostic value of routine SB in biopsy-naïve men with suspicious MRI findings remains controversial.

**Methods:**

PRIMA is a randomized, prospective, multicenter non-inferiority diagnostic accuracy trial in eight German hospitals. Biopsy-naïve men aged 50–75 years with PSA ≥ 3 ng/ml and/or suspicious DRE undergo mpMRI (PI-RADS v2.1, PI-QUAL v2). Men with PI-RADS 4–5 or PI-RADS 3 with PSA density > 0.15 are randomized 1:1 to TB only (Arm A) or TB + SB (Arm B). Persistent PI-RADS 4–5 lesions with negative biopsy undergo MRI in-bore biopsy.

**Outcomes:**

Co-primary endpoints are csPCa (ISUP ≥ 2) detection and detection of clinically insignificant cancer (ISUP 1). Secondary endpoints include patient-reported outcomes (EORTC-QLQ-C30, EPIC-26, VAS), biopsy-related complications, biopsy approach, MRI in-bore yield, AI/radiomics validation and follow-up cancer incidence.

**Sample size:**

One thousand nine hundred eight men were allocated to achieve 1590 analyzable patients (> 80% power; non-inferiority margin δ = 13%).

**Discussion:**

PRIMA will provide high-level evidence whether systematic biopsy can be safely omitted in MRI-positive biopsy-naïve men, potentially reducing diagnostic morbidity and overtreatment.

**Trial registration:**

ClinicalTrials.gov NCT04993508. Registered on 2 December 2022.

**Supplementary Information:**

The online version contains supplementary material available at 10.1186/s13063-026-09750-z.

## Introduction

### Background and rationale {6a}

In the German healthcare context, optimizing diagnostic efficiency is essential due to increasing demand for prostate cancer screening and limited reimbursement for mpMRI. Although MRI-targeted pathways have demonstrated clinical effectiveness, evidence for their cost-effectiveness and scalability in Germany remains limited. PRIMA addresses this gap by evaluating a high-quality, quality-assured MRI pathway that could reduce unnecessary biopsies and downstream treatment costs while maintaining diagnostic accuracy. Prostate cancer (PCa) is the most common solid cancer among men in Germany, with around 75,000 new diagnoses and 15,000 deaths annually, representing 25% of new male malignancies [1]. Classical diagnostic algorithms combining PSA, DRE, and 12-core SB lead to both overdetection of indolent PCa and missed csPCa [2]. Overdiagnosis results in unnecessary treatment, side effects, and psychological burden, whereas underdetection may delay curative therapy. Several prospective studies have demonstrated that TB alone provides similar detection proportions of csPCa compared with SB, while significantly lowering detection of clinically insignificant cancers [3–7]. Nonetheless, other reports suggest that SB still adds incremental diagnostic value [8–13]. The absolute additional yield of SB for csPCa detection has been estimated at 4–5% in two randomized trials and a Cochrane review [8–10], while TB alone may reduce the detection of ISUP grade group 1 cancers by up to 40% [7]. More recent data from the PROBASE trial in younger men (45–55 years) in a screening setting demonstrated that SB identified 26% additional csPCa compared with TB alone, whereas TB contributed only 6% additional csPCa [14]. Screening trials such as STHLM3-MRI [11] and Göteborg-2 [12] provided population-level insights. In STHLM3-MRI, MRI-based pathways reduced unnecessary biopsies and detection of low-grade cancers but would have missed ~1.7 csPCa cases for each avoided ISUP 1 diagnosis [11]. Göteborg-2 demonstrated comparable csPCa detection between TB-only and combined strategies, with fewer insignificant cancers in the TB-only arm (0.6% vs 1.2%), though some intermediate-risk cancers were detected only by SB [12]. Together, these studies highlight that MRI-targeted pathways can safely reduce overtreatment but may risk missing a small proportion of csPCa. Within Germany, mpMRI before biopsy is guideline-recommended [15]. However, the routine addition of SB remains under debate. The PROBASE trial, focusing on younger men (45–55 years), revealed that SB detected 26% additional csPCa compared to TB, suggesting that MRI interpretation and tumor biology may differ with age [16]. Furthermore, the PRIME trial recently demonstrated that bpMRI could suffice in older men (median age 65) [17], whereas PRIMA intentionally uses mpMRI to address potential diagnostic challenges in younger and intermediate-risk groups [18, 19]. Beyond diagnostic accuracy, PRIMA integrates patient-reported outcomes (PROs) to assess procedural burden (VAS) and quality of life (EORTC-QLQ-C30, EPIC-26). These outcomes are essential as biopsy techniques evolve from transrectal to transperineal approaches, potentially reducing infection risk and improving patient experience [13]. Quality assurance (QA) is a major element of PRIMA: all radiology centers are Q2-certified, ensuring compliance with PI-RADS v2.1 [20] and PI-QUAL v2 [21] standards, while urologists must document proficiency in ≥ 100 fusion biopsies. Despite robust international data, most trials have mixed cohorts with prior biopsy history, different MRI protocols, or lacked uniform QA. PRIMA is unique in enrolling exclusively biopsy-naïve men with well-defined MRI risk thresholds (PI-RADS 4/5 or PI-RADS 3 with PSAD > 0.15) and applying a standardized national QA framework. The study therefore aims to generate definitive evidence for or against omission of SB in MRI-positive men within a German healthcare setting.

### Objectives {7}

#### Primary objective

- To determine whether MRI/US fusion-guided targeted biopsy (TB) alone is non-inferior to combined targeted plus systematic biopsy (TB + SB) for detecting clinically significant prostate cancer (csPCa; ISUP ≥ 2) in biopsy-naïve men with suspicious mpMRI.

#### Secondary objectives

- To evaluate whether TB alone reduces overdetection of clinically insignificant PCa (ISUP 1) compared with TB + SB.
- To compare patient-reported outcomes (EPIC‑26, EORTC‑QLQ‑C30, VAS) between arms.
- To assess complication rates, biopsy avoidance due to mpMRI triage, biopsy approach (transrectal vs transperineal), MR in-bore biopsy yield, ISUP ≥ 3 cancers, stability of PI-RADS on follow-up MRI, and bpMRI vs mpMRI performance.
- To validate AI and radiomics models for PCa detection.

### Trial design {8}

PRIMA is a prospective, randomized (1:1), multicenter, non-inferiority, parallel-group diagnostic accuracy trial comparing TB alone (Arm A) with TB + SB (Arm B). Randomization is stratified by center using a secure web-based randomization module. The study includes exclusively biopsy-naïve men.

## Methods: participants, interventions, and outcomes

This protocol was prepared in accordance with the SPIRIT 2013 guidelines [22]. The completed SPIRIT checklist is provided as Additional file 1.

### Study setting {9}

The University Hospital Düsseldorf and the German Cancer Research Center (DKFZ) coordinate the trial.

Eight study sites in North Rhine-Westphalia, Germany: five university hospitals (Aachen, Cologne, Düsseldorf, Essen, Herne) and three community hospitals (Mönchengladbach, Essen-Mitte, Recklinghausen).

### Eligibility criteria {10}

#### Inclusion criteria

- Male, 50–75 years
- PSA ≥ 3 ng/ml and/or abnormal DRE
- Biopsy-naïve
- Able to provide written informed consent

#### Exclusion criteria

- Prior prostate biopsy or known PCa
- Contraindications to MRI (e.g., incompatible implants)
- Acute prostatitis
- Inability to undergo MRI or biopsy

### Who will take informed consent? {26a}

Informed consent will be obtained by a qualified study physician at the respective participating study site. Only medically trained investigators who are authorized according to national regulations and listed on the site delegation log may conduct the consent process. Participants will receive written and verbal information outlining trial procedures, potential risks and benefits, alternative diagnostic options, confidentiality provisions, and the voluntary nature of participation. Sufficient time will be provided for questions before written consent is documented. Signed consent forms will be stored securely at each site and managed according to Good Clinical Practice (GCP) requirements.

### Additional consent provisions for collection and use of participant data and biological specimens {26b}

The informed consent process includes consent for the scientific use of pseudonymized MRI imaging data and corresponding pathology results generated within the PRIMA trial. These data may be used for future ethically approved research projects related to prostate cancer diagnostics, including radiomics and artificial intelligence model development and validation. No biological specimens or physical biopsy material will be stored or used for secondary research purposes. All secondary analyses will be performed exclusively on double-pseudonymized or anonymized datasets, ensuring that no directly identifying personal information is accessible to researchers at any time.

## Interventions

### Explanation for the choice of comparators {6b}

The comparator arm (TB + SB) reflects the current standard of care recommended in national and international guidelines and widely practiced across Germany and Europe. Systematic biopsy remains routine in biopsy-naïve men because prior evidence suggests a potential incremental diagnostic yield of approximately 4–5% for clinically significant prostate cancer (csPCa) when added to targeted biopsy. Therefore, TB + SB serves as the appropriate control against which the non-inferiority of TB-only can be rigorously evaluated.

### Intervention description {11a}

#### PSA testing

PSA tests will be performed by general practitioners, office-based urologists, or participating study centers. If a confirmed PSA test is elevated (≥ 3 ng/ml), men will be screened for potential study inclusion.

#### Digital rectal examination (DRE)

DRE is not required for study inclusion. However, in case of a suspicious DRE finding, participants may be included even without elevated PSA. DRE can be performed by general practitioners, office-based urologists, or study center urologists.

#### Multiparametric MRI (mpMRI)

MRI will be acquired and reported in accordance with PI-RADS v2.1 and national guideline standards. Scanning will be performed using 1.5 T or 3 T MRI systems (< 10 years old) equipped with multi-phased array surface coils. Only scanners meeting PI-QUAL v2 score 2–3 will be approved. The mpMRI protocol includes T1w, T2w, DWI, and DCE sequences. Butylscopolamine may be administered to optimize image quality. MRI studies below predefined quality thresholds must be repeated. Each center will submit sample imaging for approval prior to patient enrollment, undergo pre-monitoring by the PRIMA core team, and receive annual on-site monitoring. All radiology departments are Q2-certified according to German Radiological Society criteria.

#### Prostate biopsy

MRI/US fusion targeted biopsies (TB) and systematic 12-core biopsies (SB) will be performed via transrectal or transperineal access under local or general anesthesia. TB consists of 4 targeted cores per lesion (up to 3 lesions). Participating urologists must demonstrate certification and ≥ 100 lifetime fusion biopsies, following a standardized QA protocol. Implementation adherence will be monitored as detailed in the quality handbook.

#### MR in-bore biopsy

For participants with PI-RADS 4–5 lesions and negative TB or TB + SB, MR in-bore biopsy will be offered after consensus reading. Needle placement will be confirmed in two imaging planes, and 2–4 cores per lesion will be acquired, with additional cores permitted if targeting is inaccurate.

#### Randomization scheme

Men with PI-RADS 4–5 or PI-RADS 3 with PSAD > 0.15 will be randomized 1:1 into Arm A (TB only) or Arm B (TB + SB).

### Criteria for discontinuing or modifying allocated interventions {11b}

Because PRIMA is a diagnostic accuracy trial without therapeutic intervention, no modification or dose adjustment of assigned study procedures is possible. However, allocated biopsy procedures may be discontinued or deferred for an individual participant under the following circumstances:

- Participant decision or withdrawal of consent at any point prior to or during the biopsy procedure, without a requirement to provide justification and without any impact on subsequent clinical care.
- Medical judgment of the responsible study physician, if performing the biopsy would expose the participant to unacceptable clinical risk (e.g., acute infection, severe bleeding disorder, anticoagulation that cannot be safely paused, or significant deterioration in health status).
- Safety considerations arising during the procedure, including uncontrolled pain, vasovagal reaction, or procedure-related complications.
- Protocol deviations mandated by emergency clinical indications, if continuation is deemed inappropriate for patient safety.

Participants who discontinue or do not complete their allocated intervention will be encouraged to remain in the study for follow-up assessments where possible, and data already collected may be included in analyses according to the predefined statistical plan (intention-to-treat principle).

### Strategies to improve adherence to interventions {11c}

As PRIMA is a diagnostic accuracy trial without therapeutic intervention, adherence is expected to be high. All participants (Arms A and B) benefit from access to high-quality mpMRI and standardized biopsy procedures at experienced centers, which is commonly perceived as advantageous care. All participants receive structured follow-up imaging and, if indicated, MR in-bore biopsy to ensure that csPCa is not missed in either study arm, reducing concerns about randomization.

Adherence will be supported through clear communication, appointment reminders, and direct contact with study nurses. Completion of scheduled procedures will be monitored through the electronic trial database, and adherence metrics will be reviewed in monthly Trial Steering Committee meetings.

### Relevant concomitant care permitted or prohibited during the trial {11d}

All standard medical care for benign prostate conditions and comorbidities is permitted during the trial. Concomitant medications, including anticoagulants or antiplatelet agents, may be continued according to institutional biopsy safety protocols and national guidelines; temporary adjustment for biopsy procedures is allowed according to routine clinical standards.

External mpMRI examinations or prostate biopsies are not prohibited. However, they are strongly discouraged during trial participation because the study protocol provides comprehensive, high-quality diagnostic assessment—including structured imaging follow-up and MR in-bore biopsy where indicated—designed to minimize risk of missed csPCa. If external diagnostic procedures are nevertheless performed due to urgent clinical need or patient request, they must be documented in the trial database and the participant may be withdrawn if protocol integrity is compromised.

### Provisions for post-trial care {30}

All participants are covered by a clinical trial insurance policy arranged prior to the start of recruitment, which provides compensation for any harm directly related to trial participation, including biopsy-associated complications. Standard clinical follow-up and treatment of any medical conditions identified during the trial will continue within routine healthcare services. No additional post-trial interventions are planned, as PRIMA is a diagnostic trial without therapeutic assignment, and all participants receive guideline-based clinical care independent of study completion.

### Outcomes {12}

#### Co-primary outcomes

- Detection of clinically significant prostate cancer (csPCa; ISUP ≥ 2)

Defined as the presence of csPCa on histopathology at initial biopsy (measure: histopathological assessment; metric: binary yes/no). Results will be expressed as the proportion of participants with csPCa in each study arm (method of aggregation: proportion), assessed at index biopsy (timepoint).

- Detection of clinically insignificant prostate cancer (ISUP 1)

Defined as the presence of ISUP grade group 1 cancer on histopathology at initial biopsy (measure: histopathological assessment; metric: binary yes/no). Results will be expressed as the proportion of participants with ISUP 1 cancer in each study arm, assessed at index biopsy.

#### Secondary outcomes

- Patient-reported outcomes (EPIC-26, EORTC-QLQ-C30, VAS)

Measured using validated questionnaires (metric: continuous scores), analyzed as mean changes over time and between groups (aggregation: mean differences), assessed at baseline, 30 days, and 12 months.

- Biopsy-related complications

Defined as adverse events within 30 days post-biopsy (measure: clinical assessment; metric: categorical/binary), summarized as proportions by study arm, assessed at 30 days post-procedure.

- Biopsy avoidance due to mpMRI triage

Defined as the proportion of participants not undergoing biopsy due to non-suspicious mpMRI findings (PI-RADS 1–2 or PI-RADS 3 with PSAD ≤ 0.15), reported as proportion, assessed at screening.

- Diagnostic performance of DRE

Defined as the association between DRE findings and biopsy outcomes (metric: sensitivity, specificity), analyzed cross-sectionally at baseline and biopsy.

- Biopsy approach (transrectal vs. transperineal)

Compared in terms of pain (VAS score) and infection rates (binary), summarized as mean differences and proportions, assessed within 30 days post-biopsy.

- MR in-bore biopsy yield

Defined as detection of csPCa in participants undergoing MR in-bore biopsy after negative or ISUP 1 results (metric: binary), reported as proportion, assessed within 3 months post-biopsy.

- Detection of higher-grade cancer (ISUP ≥ 3)

Defined as presence of ISUP ≥ 3 cancer on histopathology (metric: binary), reported as proportion, assessed at index biopsy.

- Comparison of bpMRI vs mpMRI

Retrospective comparison of detection performance (metric: paired binary outcomes), analyzed using constrained maximum likelihood methods, assessed at baseline imaging.

- Change in PI-RADS score

Defined as change in PI-RADS category between baseline and follow-up MRI (metric: ordinal change), reported as proportions of up- or downgrading, assessed at 12 months.

- Index-lesion-only analysis

Detection of csPCa based on index lesion targeting only (metric: binary), reported as proportion, assessed at index biopsy.

- AI and radiomics validation

Diagnostic performance of AI-based models (metric: AUC, sensitivity, specificity), analyzed retrospectively, assessed using baseline imaging and biopsy outcomes.

### Participant timeline {13}

**Fig. 1:**
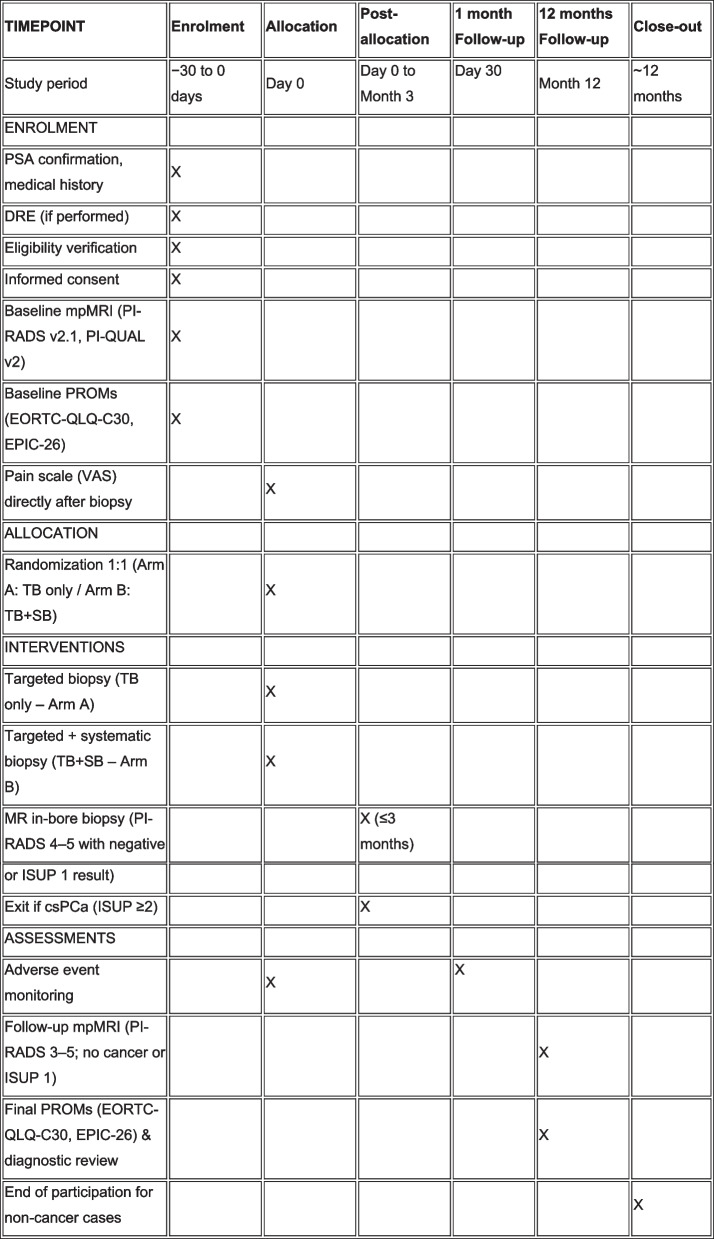
SPIRIT schedule of enrollment, interventions and assessments for the PRIMA Trial. SPIRIT figure illustrating the structured timeline of trial procedures including screening, baseline imaging, randomization, biopsy, MR in-bore biopsy (if indicated), safety follow-up at 30 days, 12-month follow-up imaging and PROMs, and end-of-study data lock

The participant timeline includes screening, enrollment, baseline assessments, randomization, biopsy procedures, and structured follow-up over 12 months. Only men with mpMRI findings of PI-RADS 4–5 or PI-RADS 3 with PSA density > 0.15 proceed to randomization. Follow-up includes standardized complication reporting, patient-reported outcome assessments, and repeat MRI depending on biopsy results (see Fig. 1).

### Sample size {14}

#### Assessment for eligibility

Men aged 50–75 with PSA elevation (≥ 3 ng/ml) and/or abnormal DRE. To be allocated to trial: *n* = 1908 (including 20% calculated loss). To be analyzed: 477 patients per randomized arm A/B (PI-RADS 4/5 and PI-RADS 3 PSAD > 0.15), resulting in *N* = 2 × 477/0.6 = 1590.

#### Sample size

The sample size is derived from the following expected PI-RADS distribution (own institutional data and PRECISION trial data [8, 23]): 25% PI-RADS 1–2, 25% PI-RADS 3 (15% PSAD ≤ 0.15 and 10% PSAD > 0.15), 30% PI-RADS 4, and 20% PI-RADS 5. Thus, the prevalence of PI-RADS 4/5 and PI-RADS 3 PSAD > 0.15 is estimated at 60%. The estimated prevalence of csPCa in this cohort is 50%, and the detection of low-grade PCa (ISUP 1) is expected to be 15%.

Based on prior evidence, targeted biopsy alone is expected to reduce ISUP 1 detection by at least 40% [7], and SB adds approximately 5% absolute incremental csPCa detection according to randomized trials and a Cochrane meta-analysis [9, 10, 13, 14]. Non-inferiority of TB alone compared with TB + SB will be evaluated by testing H0: Δ0 = πTB − πSB + TB ≤ − δNI, with a non-inferiority margin of δNI = 13%. The non-inferiority margin of 13% was chosen based on prior evidence demonstrating an approximate 4–5% incremental detection of clinically significant prostate cancer with systematic biopsy, allowing for a conservative margin that maintains clinical relevance. Assuming πSB + TB = 50% and a true difference of Δ = − 5%, 477 patients per arm ensure 80% power at one-sided α = 0.05.

Superiority in avoiding ISUP 1 diagnoses will be tested under H0: πTB/πSB + TB ≥ 1. With πSB + TB = 15% and a true reduction ratio to 60%, a sample size of 460 patients per arm ensures 88% power at *α* = 0.05.

Therefore, 1590 analyzable patients are required (2 × 477/0.6 = 1590), and accounting for 20% dropout, approximately 1908 must be allocated within 3 years.

#### Feasibility

More than 2500 fusion biopsies are performed annually across participating centers. Recruitment expectations: 1908 within 36 months (636/year; ~64 per center/year; 51 analyzable). PRIMA recruitment does not depend solely on community referrals but is expected to be supported due to advantages for patients such as cost-covered mpMRI and biopsy avoidance in negative imaging cases.

### Recruitment {15}

Eight established centers perform > 2500 fusion biopsies annually, providing sufficient recruiting capacity. Stopping rules are applied if < 50% first-year enrollment or < 70% by year two.

## Assignment of interventions: allocation

### Sequence generation {16a}

Participants will be randomized in a 1:1 allocation ratio to either Arm A (targeted biopsy only) or Arm B (targeted + systematic biopsy). Randomization will use computer-generated permuted blocks of variable size and will be stratified by study center to ensure balance across recruitment sites.

### Concealment mechanism {16b}

Allocation concealment will be ensured through a secure web-based automated randomization system integrated with the electronic data capture software. The system will release the assignment only after participant eligibility is confirmed, preventing foreknowledge or prediction of allocation. Treating investigators and study personnel remain blinded to allocation until the moment it is irreversibly generated.

### Implementation {16c}

Randomization will be initiated by authorized study personnel at each participating study site via the secure randomization portal. Assignment to either intervention arm will then be automatically recorded within the trial documentation system. The coordinating investigator and monitoring team will not have access to the sequence generation algorithm or block sizes.

## Assignment of interventions: blinding

### Who will be blinded {17a}

Clinical teams cannot be blinded due to procedural differences between study arms. Radiological assessment (including PI-RADS scoring) is performed prior to randomization and is therefore inherently blinded to treatment allocation. Biostatistical analyses will be conducted on pseudonymized datasets, with treatment allocation unmasked only after database lock, following finalization of the statistical analysis plan.

### Procedure for unblinding if needed {17b}

Not applicable, as no blinding of participants or treating clinicians is implemented; therefore, no unblinding procedures are required.

## Data collection and management

### Plans for assessment and collection of outcomes {18a}

Outcome, baseline, and follow-up data will be collected using standardized electronic case report forms (eCRFs) implemented in a centralized, web-based clinical data management platform hosted by the German Cancer Research Center (DKFZ), Heidelberg. Data collection is performed by trained study personnel at participating sites following a standardized operations manual and quality assurance procedures.

Clinical, imaging, procedural, and pathological outcome data will be documented based on predefined categories and automated plausibility and range checks to ensure accuracy and completeness. Patient-reported outcome measures (PROMs) will be collected using validated German language versions of standardized instruments, including EPIC-26, EORTC-QLQ-C30 and VAS scales. Questionnaires will be completed on paper or electronically and transferred to eCRFs using double-check verification if manually entered. Radiological findings will be reported according to PI-RADS v2.1, and MR image quality will be rated using PI-QUAL v2. All pathologic assessments follow ISUP 2014 Gleason grading criteria. Detailed eCRF structures and data collection forms are available in the study operations handbook.

### Plans to promote participant retention and complete follow-up {18b}

Participant retention in PRIMA is supported by clear communication of the clinical relevance of structured imaging follow-up and by scheduling support from study nurses at each site. Reminder systems, including scheduled telephone, electronic or postal notifications, will be used to encourage attendance at follow-up visits, particularly the 30-day safety visit and the 12-month mpMRI assessment. Missed appointments will be actively followed up by site investigators or study nurses to re-schedule assessments where feasible.

If participants discontinue allocated biopsy procedures, routinely collected clinical data—including follow-up MRI results, histopathology reports, adverse events and patient-reported outcomes—will continue to be collected unless consent is formally withdrawn. Reasons for withdrawal or protocol deviation will be documented in the electronic trial database.

For participants lost to follow-up, electronic hospital records may be reviewed to identify biopsy results, MRI findings, or cancer diagnoses when permitted by local data-protection regulations. Participants diagnosed with csPCa (ISUP ≥ 2) will exit the trial and enter guideline-based standard-of-care treatment; data collection until the time of exit remains part of the primary analysis.

### Data management {19}

All data management processes follow a data protection plan developed under the supervision of the Department of Data Protection at the German Cancer Research Center (DKFZ). Data are entered into a secure, password-protected database using pseudonymized participant identifiers. Automated validation rules, range checks, audit trails, and real-time query resolution support data quality. Access rights are role-based and restricted to authorized study staff. Monitoring of data quality is performed by the coordinating center and includes remote and on-site review. Full data management procedures are documented in the trial data management handbook.

### Confidentiality {27}

Personal identifying information is stored separately from clinical study data. Pseudonymization is applied via site-specific alias keys accessible only to authorized investigators. MRI image data are stored securely using the same pseudonymization structure. All paper documents are kept in locked archives at study sites. Data transmission occurs via encrypted channels. Data will be retained for a minimum of 10 years after study completion in accordance with German regulations and GDPR. No personally identifiable data will be disclosed in publications.

### Plans for collection, laboratory evaluation and storage of biological specimens for genetic or molecular analysis in this trial/future use {33}

No biological specimens for genetic or molecular analysis are collected as part of the PRIMA main trial.

## Statistical methods

### Statistical methods for primary and secondary outcomes {20a}

The primary analysis will compare the detection proportion of clinically significant prostate cancer (csPCa; ISUP ≥ 2) between Arm A (TB only) and Arm B (TB + SB) using a non-inferiority framework. Non-inferiority for csPCa detection will be assessed based on the absolute difference in detection proportions (π_TB − π_TB + SB), evaluated using the likelihood score test by Gart and Nam for binomial parameters at a one-sided α level of 0.05. The non-inferiority margin is δ = 13%. Superiority testing for the secondary co-primary endpoint (detection of clinically insignificant PCa, ISUP 1) will be evaluated based on the ratio of detection proportions (π_TB/π_TB + SB) using a one-sided α level of 0.05. All hypothesis tests apply a fixed global significance level of *α* = 0.05.

Secondary outcomes—including biopsy-related complications, biopsy avoidance, diagnostic performance measures, and biopsy approach comparisons—are defined in the Outcomes section according to domain, measurement, metric, method of aggregation, and timepoint. Biopsy-related complications will be summarized as proportions by study arm at 30 days post-procedure. Biopsy avoidance will be reported as the proportion of participants not undergoing biopsy following mpMRI triage at screening. Diagnostic performance outcomes (e.g., DRE, bpMRI vs mpMRI) will be evaluated using standard accuracy measures (e.g., sensitivity, specificity) where applicable.

All detection outcomes are defined as proportions (number of participants with the outcome divided by the number of participants undergoing biopsy in the respective arm). Secondary outcomes will be analyzed descriptively and, where appropriate, using inferential statistical methods.

Patient-reported outcomes (EPIC-26, EORTC-QLQ-C30, VAS) will be analyzed using mixed models for repeated measures (MMRM), including treatment arm, timepoint, and baseline values as covariates.

A detailed statistical analysis plan (SAP) will be finalized and archived prior to database lock and prior to any unblinded analyses.

### Interim analyses {21b}

No interim efficacy analyses are planned. Recruitment feasibility will be monitored, and predefined stopping criteria apply solely for insufficient enrollment. Safety data will be reviewed periodically by the coordinating investigators. Only the principal investigators and the independent monitoring committee will have access to aggregated safety data, and they will make decisions regarding trial continuation or modifications if necessary.

### Methods for additional analyses (e.g., subgroup analyses) {20b}

Pre-specified subgroup analyses will explore whether the effect of targeted biopsy alone versus combined biopsy on the detection proportion of clinically significant prostate cancer (csPCa; ISUP ≥ 2) varies across clinically relevant strata, assessed at index biopsy.

Planned subgroup analyses include:

- PI-RADS category: PI-RADS 4 vs PI-RADS 5
- PSA density (PSAD): ≤ 0.15 vs > 0.15–0.20 vs > 0.20
- Biopsy approach: transrectal vs transperineal
- Prostate volume: < 60 ml vs 60–100 ml vs > 100 ml
- Age categories: 50–60, 61–70, 71–75 years
- Number of MRI lesions: single index lesion vs multiple lesions
- MRI quality: PI-QUAL 2 vs PI-QUAL 3
- Follow-up imaging outcome: stable vs up- or downgraded PI-RADS at 12 months
- Histopathology outcome: ISUP 1 only vs no cancer on initial biopsy

Interaction terms will be evaluated using logistic regression models to assess effect modification on the primary endpoint. All subgroup analyses will be exploratory, hypothesis-generating, and interpreted descriptively without correction for multiplicity.

### Methods in analysis to handle protocol non-adherence and any statistical methods to handle missing data {20c}

The primary analysis will follow the intention-to-treat (ITT) principle, including all randomized participants regardless of protocol deviations. A secondary per-protocol (PP) analysis will exclude patients with major deviations (e.g., incorrect biopsy allocation, inadequate imaging quality). Missing outcome data will be addressed primarily through multiple imputation techniques assuming missing at random. Sensitivity analyses include complete-case analysis and worst-case imputation for key endpoints.

### Plans to give access to the full protocol, participant level-data and statistical code {31c}

The full study protocol and statistical analysis plan (SAP) will be publicly accessible upon publication of the main results. Participant-level pseudonymized datasets and statistical code will be made available upon reasonable request to the corresponding author after completion of primary analyses and institutional review, in accordance with GDPR and data governance regulations.

## Oversight and monitoring

### Composition of the coordinating center and trial steering committee {5d}

The coordinating center for the PRIMA trial is located at the University Hospital Düsseldorf and is responsible for day-to-day operational management, site communication, regulatory coordination, investigator training, and monitoring oversight. The German Cancer Research Center (DKFZ), Heidelberg, oversees biostatistical infrastructure, including randomization processes, data management systems, and final statistical analysis.

The Trial Steering Committee (TSC) provides scientific oversight, monitors trial progress, evaluates protocol adherence, resolves methodological and logistical issues, and approves protocol amendments. The TSC meets monthly, or more frequently if required. The committee is composed of the Principal Investigators and all co-applicants (see also 5a). The committee receives operational support from the coordinating center trial office, which manages cross-site communication, documentation control, quality assurance processes, and adherence to trial timelines.

### Composition of the data monitoring committee, its role and reporting structure {21a}

A Data Monitoring Committee is not required given the low-risk, diagnostic nature of the trial. Independent data and safety monitoring will be performed by the German Cancer Research Center according to Good Clinical Practice standards.

### Adverse event reporting and harms {22}

Adverse events (AEs) and serious adverse events (SAEs) related to biopsy procedures will be collected systematically from the time of intervention up to 30 days post-biopsy and documented in the electronic case report forms. In addition, specific complications will be actively assessed at 30 days using a structured patient survey, including sepsis, hospitalization, bleeding, urinary retention, persistent pain, hemospermia, and urinary tract infection.

SAEs will be reported to the sponsor and relevant ethics committees in accordance with national regulatory requirements. All events will be reviewed by the coordinating investigators.

In publications, all collected adverse events will be reported descriptively by study arm, including frequencies and severity grading. Serious adverse events and clinically relevant complications will be reported in detail to ensure transparent and comprehensive safety reporting.

The frequency and nature of biopsy-related complications are predefined secondary outcomes.

### Frequency and plans for auditing trial conduct {23}

Independent monitoring will be performed according to a risk-adapted monitoring plan and performed by qualified personnel independent of the investigators and sponsor. On-site monitoring visits will be conducted at least annually, with additional remote monitoring for consent documentation, endpoint verification, and source data consistency. Monitoring activities include verification of adherence to the protocol, data accuracy, informed consent procedures, and reporting of adverse events. Audit results and corrective actions will be documented and communicated to participating centers and the TSC.

### Plans for communicating important protocol amendments to relevant parties (e.g., trial participants, ethical committees) {25}

Any important protocol modifications (e.g., changes to eligibility criteria, sample size, outcomes, or analytic strategy) will be communicated to all relevant stakeholders, including participating investigators, ethics committees, regulatory authorities, trial registries, and study participants, where applicable. Updated protocol versions will be resubmitted to registries (ClinicalTrials.gov) and annotated within future publications. Changes will also be documented within the electronic trial master file and circulated through official newsletters and site investigator meetings.

### Dissemination plans {31a}, {31b}

Results will be disseminated through peer-reviewed open-access publications, scientific conferences, and patient stakeholder organizations irrespective of study outcome. Authorship will follow ICMJE guidelines. The full protocol, statistical code, and de-identified dataset will be made available upon reasonable request after publication.

## Discussion

The PRIMA study is designed to resolve a central question in modern prostate diagnostics: is systematic biopsy still required for biopsy-naïve men in the era of high-quality mpMRI and fusion-guided TB? Current evidence remains mixed. Trials such as PRECISION [8], MRI-FIRST [9], and 4-M [10] confirmed that mpMRI-guided TB achieves high csPCa detection, but residual incremental detection from SB remains. Screening trials like STHLM3-MRI [11] and Göteborg-2 [12] further emphasized that omitting SB reduces overdetection but carries a marginal risk of missing csPCa. Thus, guidelines differ internationally, and a definitive, high-quality randomized trial within a controlled QA environment is urgently needed. Compared to prior studies, PRIMA features several distinguishing elements. First, strict preselection limits biopsy to men with PI-RADS 4/5 or PI-RADS 3 with PSAD > 0.15, consistent with German guideline recommendations [15, 24]. This enriched population minimizes unnecessary biopsies while maintaining csPCa yield. Second, mpMRI is mandatory rather than optional or replaced by bpMRI, ensuring maximal lesion characterization, especially in younger cohorts where imaging interpretation is more challenging [18, 19]. Third, uniform QA through PI-QUAL and operator certification standardizes both imaging and biopsy performance across all sites. Finally, inclusion of PROs allows holistic assessment of diagnostic burden, patient satisfaction, and complication profiles. The study also integrates advanced research objectives such as AI and radiomics model validation. By linking imaging features, histopathology, and PRO data, PRIMA aims to establish predictive algorithms for csPCa and quality-of-life outcomes, providing a platform for future personalized diagnostics. Expected benefits include reduced biopsy numbers, lower infection rates, and fewer ISUP 1 detections—leading to substantial health-economic savings [2, 3, 7]. Conversely, if SB omission compromises csPCa detection, results will clarify which subgroups (e.g., age, prostate volume, PI-RADS 3) still benefit from combined sampling. The standardized design and large sample will support guideline updates, reimbursement decisions, and possible nationwide implementation of MRI-only pathways if non-inferiority is demonstrated. Lastly, the PRIMA trial will further assess under which conditions and to what extent MRI in-bore biopsy constitutes a valid and safe approach within a targeted biopsy-only paradigm [25–28]. Limitations include potential selection bias toward academic centers with strong MRI expertise and limited generalizability to less-specialized institutions. Nevertheless, PRIMA’s rigorous QA, centralized monitoring, and defined endpoints ensure validity and reproducibility. Its findings will directly influence diagnostic policy, reduce overtreatment, and optimize early detection of clinically meaningful prostate cancer in Germany.

## Trial status

Protocol version v1.0, dated 15 January 2026. Recruitment is planned for 15 April 2026 and is expected to continue for 36 months. The anticipated completion of recruitment is 15 April 2029, with final follow-up ending approximately April 2030 and database lock projected for October 2030.

This article reports the initial approved protocol prior to recruitment. No participants have been enrolled at the time of manuscript submission.

## Supplementary Information

Additional file 1: Informed consent.

## Data Availability

No datasets were generated or analysed during the current study.

## Abbreviations

AE: Adverse event
AI: Artificial intelligence
csPCa: Clinically significant prostate cancer
DCE: Dynamic contrast-enhanced MRI
DMC: Data Monitoring Committee
DRE: Digital rectal examination
DWI: Diffusion-weighted imaging
EPIC-26: Expanded Prostate Cancer Index Composite (26-item)
eCRF: Electronic case report form
GDPR: General Data Protection Regulation
GCP: Good Clinical Practice
ICMJE: International Committee of Medical Journal Editors
ISUP: International Society of Urological Pathology
ITT: Intention-to-treat
mpMRI: Multiparametric magnetic resonance imaging
MR: Magnetic resonance
PROM: Patient-reported outcome measure
PI-RADS: Prostate Imaging Reporting and Data System
PI-QUAL: Prostate Imaging Quality Score
PCa: Prostate cancer
PSA: Prostate-specific antigen
PSAD: PSA density
QA: Quality assurance
SAP: Statistical analysis plan
SB: Systematic biopsy
SAE: Serious adverse event
TB: Targeted biopsy
TR: Transrectal
TP: Transperineal
TSC: Trial Steering Committee
VAS: Visual analog scale
