# Supplementary material for: PRIMA: randomized prospective multicenter non-inferiority study for primary diagnosis of clinically significant PRostate cancer by PSA and MR IMAging—study protocol for a randomized diagnostic accuracy trial
Source: Trials. 2026 Apr 25;27:320. doi: 10.1186/s13063-026-09750-z (PMC13109873; doi:10.1186/s13063-026-09750-z)
Supplement: Supplementary file 1 — Additional file 1: Informed consent. [file 13063_2026_9750_MOESM1_ESM.docx]

**Appendices**

**Informed consent original:**

**PRIMA-Studie – Einwilligungserklärung zur Studienteilnahme**

**Randomisierte prospektive multizentrische Studie zur Primärdiagnose von klinisch signifikantem Prostatakrebs durch PSA und MRT-Bildgebung**

Sehr geehrter Patient,
vielen Dank für Ihr Interesse und Ihre Bereitschaft zur Teilnahme an der PRIMA-Studie. In den folgenden Abschnitten der Einwilligungserklärung können Sie Ihre Zustimmung zur Studienteilnahme und zur Verarbeitung Ihrer Daten im Rahmen der PRIMA-Studie erklären.
Die Teilnahme an dieser Studie erfolgt freiwillig. Sie können einzelne oder alle Einwilligungen jederzeit, ohne Angabe von Gründen und ohne Nachteile für Ihre medizinische Behandlung, schriftlich oder mündlich widerrufen.

Bitte lesen Sie alle Informationen sorgfältig und stellen Sie beim Aufklärungsgespräch gegebenenfalls Fragen. Wir bitten Sie, Ihre Entscheidungen anzukreuzen und das Formular zu unterschreiben.

1. Teil: Allgemeine Studienteilnahme

Die Zustimmung zu den folgenden Punkten ist Voraussetzung für die Teilnahme an der Studie.

Ich willige ein, an der PRIMA-Studie zur Primärdiagnose von klinisch signifikantem Prostatakrebs mittels PSA-Test, digital-rektaler Untersuchung (DRE), multiparametrischer Magnetresonanztomographie (mpMRT) und gezielter Prostatabiopsie teilzunehmen. Ich bestätige, dass ich die schriftliche Studienteilnehmer-Information und eine Kopie dieser Einwilligungserklärung erhalten und verstanden habe.
Ich bin bereit, die vereinbarten Untersuchungstermine im Studienzentrum wahrzunehmen. Ich bin mir bewusst, dass ich nicht an der Studie teilnehmen kann, wenn ich eine MRT-Untersuchung oder die Prostatabiopsie ablehne oder keine Fragen zu meiner Person oder meinem Gesundheitszustand beantworten möchte.

Ich willige in die Erhebung, Übermittlung, Speicherung und Verarbeitung meiner personenbezogenen Daten, insbesondere meiner Gesundheitsdaten, am Studienzentrum sowie am Deutschen Krebsforschungszentrum (DKFZ) gemäß der Studienteilnehmer-Information ein.

Einwilligung 1.1: ☐ ja ☐ nein

Ich willige ein, dass im Rahmen der MRT-Untersuchung Kontrastmittel verabreicht wird, sofern keine Kontraindikationen vorliegen. Über mögliche Risiken wurde ich aufgeklärt.
Ich bin mit der pseudonymisierten Weitergabe, Speicherung und Verarbeitung meiner MRT-Bilddaten zur Qualitätssicherung und wissenschaftlichen Auswertung an das Institut für Diagnostische und Interventionelle Radiologie am Universitätsklinikum Düsseldorf sowie an das DKFZ und mit einer anonymisierten Weitergabe an wissenschaftliche Kooperationspartner einverstanden.

Einwilligung 1.2: ☐ ja ☐ nein

Ich bin mit der Entnahme einer Prostatagewebeprobe (Biopsie) entsprechend dem Ergebnis der mpMRT einverstanden. Mir ist bekannt, dass ein Zufallsverfahren (50:50-Chance) entscheidet, ob zusätzlich systematische Gewebeproben entnommen werden.
Ich wurde über die Risiken einer Prostatabiopsie aufgeklärt. Ich bin außerdem mit einer MRT-gesteuerten in-bore-Biopsie einverstanden, falls bei hochgradig auffälligem MRT-Befund kein klinisch signifikanter Prostatakrebs nachweisbar ist.

Einwilligung 1.3: ☐ ja ☐ nein

Ich willige ein in eine erneute Kontaktaufnahme zur Besprechung der Ergebnisse sowie zur Einladung zur Nachuntersuchung (nach ca. 12 Monaten). Ich bin damit einverstanden, Fragebögen zu meiner medizinischen Vorgeschichte und Lebensqualität auszufüllen.

Einwilligung 1.4: ☐ ja ☐ nein

👉 Ohne die Einwilligungen 1.1–1.4 kann ich nicht an der Studie teilnehmen.

2. Teil: Optionale zusätzliche Aspekte

Die nachfolgenden Punkte sind nicht verpflichtend für die Studienteilnahme, aber von hoher Bedeutung für die wissenschaftliche Auswertung.

2.1 Kontaktaufnahme zu zukünftigen Studien

Ich willige ein, erneut kontaktiert zu werden, um über neue Studien informiert zu werden. Eine Teilnahme an weiteren Studien erfordert eine gesonderte Einwilligung.

Einwilligung 2.1: ☐ ja ☐ nein

2.2 Entbindung von der ärztlichen Schweigepflicht

Ich willige ein, dass mein Studienzentrum bei meinem Hausarzt bzw. behandelnden Ärztinnen/Ärzten Diagnosen und Behandlungsdaten anfordern darf, die für die Studie relevant sind, und hierzu Name, Geburtsdatum und Anschrift übermittelt werden.
Im Falle meines Todes dürfen Informationen über Todesursache und Umstände eingeholt werden.
Ich entbinde meine behandelnden Ärztinnen/Ärzte von ihrer Schweigepflicht.

Einwilligung 2.2: ☐ ja ☐ nein

2.3 Datenabgleich mit Krebsregistern

Ich willige ein, dass Daten aus dem Krebsregister zur Studie übermittelt und verarbeitet werden dürfen, und dass relevante Studiendaten an die Krebsregister gemeldet werden.

Einwilligung 2.3: ☐ ja ☐ nein

2.4 Melderegisterabfrage

Ich willige ein, dass meine Daten aus dem Melderegister abgefragt und verarbeitet werden dürfen.

Einwilligung 2.4: ☐ ja ☐ nein

2.5 Gesundheitsamt-Abfrage

Ich willige ein, dass im Todesfall relevante Informationen beim Gesundheitsamt eingeholt werden dürfen.

Einwilligung 2.5: ☐ ja ☐ nein

Einwilligung zur Datenverarbeitung (Datenschutz gemäß DSGVO)

Ich bin damit einverstanden, dass im Rahmen der PRIMA-Studie personenbezogene und Gesundheitsdaten gemäß Art. 6 Abs.1 lit. a und Art. 9 Abs.2 lit. a DSGVO verarbeitet werden.

Ich willige ein, dass meine Studien- und MRT-Daten zu Studienzwecken verarbeitet und pseudonymisiert übermittelt werden an:
a) die Studiendatenbank des DKFZ
b) das Institut für Diagnostische und Interventionelle Radiologie, Universitätsklinikum Düsseldorf
c) anonymisiert an wissenschaftliche Dritte, ggf. auch in Länder außerhalb der EU

Personenidentifizierende Daten (Name, Kontaktdaten) dürfen weitergegeben werden an:
a) DKFZ – Studienorganisation
b) Institut für Diagnostische und Interventionelle Radiologie

Ich verstehe, dass:

- meine Daten nach Studienende 10 Jahre gespeichert und danach gelöscht werden,
- autorisierte Monitore und Auditoren Einsicht nehmen können,
- alle Auswertungen nur pseudonymisiert erfolgen,
- die Veröffentlichung ausschließlich aggregierte Daten enthält.

Ich kann meine Einwilligung jederzeit ohne Nachteile widerrufen und entscheiden, ob bereits erhobene Daten gelöscht oder weiterverwendet werden dürfen.

Einschränkung der Datennutzung (optional):

Erklärungen und Unterschriften

Ich habe die Informationen verstanden und bin mit der wissenschaftlichen Verwendung meiner Daten einverstanden. Ich habe die Versicherungspolice inklusive AVB erhalten.

Studienteilnehmer (Name in Druckbuchstaben): ________________________
Datum: ____________________ Unterschrift: ______________________

Aufklärende Person (Name in Druckbuchstaben): ________________________
Ich habe den Studienteilnehmer über die Studie, Ziele, Abläufe und Risiken aufgeklärt und Kopien der Dokumente übergeben.

Datum: ____________________ Unterschrift: ______________________

**Informed consent translated:**

**PRIMA Study – Patient Informed Consent Form**

**Randomized prospective multicenter study for primary diagnosis of clinically significant prostate cancer by PSA and MR imaging**

Dear Patient,

Thank you for your interest and willingness to participate in the PRIMA study. In the following sections of the informed consent form, you may declare your consent to study participation and to the requested data processing required within the PRIMA study. You may withdraw individual or all consents at any time, without providing reasons and without any disadvantages to you. Participation in the study is voluntary. Please read all information carefully and ask any questions during the consultation process.
We kindly ask you to complete this form, indicate your decisions by ticking the relevant boxes, and sign the form.

1. General consent for study participation

Consent to the following points is a prerequisite for participation in the study.

I consent to participating in the PRIMA study on primary diagnosis of clinically significant prostate cancer through PSA testing, digital rectal examination (DRE), multiparametric magnetic resonance imaging (mpMRI), and targeted prostate biopsy, and I confirm that I have received and understood the written participant information and a copy of this informed consent form. I agree to attend planned study appointments at my study center. I understand that I cannot participate in the study if I decline MRI examination or prostate biopsy. I acknowledge that I also cannot participate if I decline to answer questions about my medical condition.

I consent to the collection, transfer, storage, and processing of my personal data, including health data, at the study center and at the German Cancer Research Center (DKFZ), as described in the participant information.

Consent 1.1.: ☐ yes ☐ no

I consent to the administration of contrast agent during MRI examinations unless medical contraindications exist. I have been informed about potential risks.
I also consent to pseudonymized transfer, storage and analysis of my MRI image data for quality assurance and scientific evaluation at the Institute of Diagnostic and Interventional Radiology, University Hospital Düsseldorf, and DKFZ, and to anonymized transfer to further scientific cooperation partners.

Consent 1.2.: ☐ yes ☐ no

I consent to prostate tissue biopsy according to the MRI result as described in the participant information.
I understand that, in the case of biopsy, a randomization process (50:50 chance) will determine whether an additional systematic biopsy is performed. I am aware of the risks associated with prostate biopsy. I additionally consent to MRI in-bore biopsy if a highly suspicious MRI finding persists but no clinically significant prostate cancer is detected in the biopsy sample.

Consent 1.3.: ☐ yes ☐ no

I consent to being contacted again by my study center, including review of examination results and invitations for repeat examination (one-year follow-up). I consent to completing additional questionnaires related to medical history and quality of life.

Consent 1.4.: ☐ yes ☐ no

Without consent to items 1.1–1.4, participation in the study is not possible.

2. Additional aspects of study participation

The following optional aspects support scientific evaluation but are not mandatory for participation. Please indicate your decision:

2.1. Contact for information about future studies

I consent to being contacted regarding new research projects and clinical studies. I understand that separate consent is required for participation in such projects.

Consent 2.1.: ☐ yes ☐ no

2.2. Release from medical confidentiality

I consent that my study center may request diagnostic and treatment data related to existing or newly occurring conditions from my general practitioner and/or other treating physicians. For this purpose, my name, date of birth, and address may be transmitted. In the event of my death, information on cause and circumstances may also be requested.
I hereby release my treating physicians from their duty of confidentiality in this regard.

Consent 2.2.: ☐ yes ☐ no

2.3. Cancer registry data query

I consent to the retrieval and processing of cancer registry data related to me. I also consent to pseudonymized transfer of relevant clinical information to the cancer registry.

Consent 2.3.: ☐ yes ☐ no

2.4. Residents’ registration office data query

I consent to the retrieval and processing of address data from the local residents’ registration office.

Consent 2.4.: ☐ yes ☐ no

2.5. Public health authority inquiry

In the event of my death, I consent to the retrieval and use of relevant information from the public health authority.

Consent 2.5.: ☐ yes ☐ no

Data Protection Consent

I am aware that, in the context of conducting the PRIMA study, personal data will be processed according to legal data protection requirements and based on my voluntary and explicit consent (Art. 6(1)(a) and Art. 9(2)(a) GDPR). My consent includes processing of special categories of personal data, particularly health data.

I consent that personal data collected during the PRIMA study, including MRI image data, may be processed for the purposes described in the participant information and transferred in pseudonymized form to:

a) The study database at the German Cancer Research Center (DKFZ)
b) The Institute of Diagnostic and Interventional Radiology, University Hospital Düsseldorf
c) Third parties for scientific purposes in anonymized form, including possible transfer to countries with lower data protection standards. No unencrypted personal data will be shared.

I consent that my identifiable personal data (name and contact details) may be transferred to:
a) DKFZ study database
b) Institute of Diagnostic and Interventional Radiology, University Hospital Düsseldorf

I understand that my data will be stored for 10 years after study completion and then deleted. Study monitors or auditors may inspect study documentation for quality assurance. I release study physicians from medical confidentiality for this purpose.

I understand that I may withdraw consent at any time for the future without disadvantage. I may choose after withdrawal whether already collected data may continue to be used or should be deleted.

Responsible institution for data processing

German Cancer Research Center (DKFZ), Im Neuenheimer Feld 280, 69120 Heidelberg
Data Protection Officer: datenschutz@med.uni-duesseldorf.de

I wish to restrict the use of my data for other/future research as follows (optional):

Participant Declaration

I have understood the conditions, all questions were answered, and I consent to the scientific use of my data. I have received a copy of the insurance policy including conditions.

Participant (printed name): _______________________________
Date: _____________________ Signature: ______________________

Investigator Declaration

I have informed the participant about study purpose, procedures, and risks. The participant has received a copy of the participant information, informed consent form, and insurance policy.

Investigator (printed name): _______________________________
Date: _____________________ Signature: ______________________
